# The Carriage of Cysts of Entamoeba, Histolytica, Protozoa, and Eggs, by House-Flies

**Published:** 1918-05

**Authors:** 


***The Carriage of Cysts of Entamoeba, Histolytica, Protozoa, and Eggs, by House-Flies.*** By Lieut.-Col. C. M. WENYON and Capt. F. W. O'CONNOR. Abs. from *Journ. R. A. M. C.*, May, 1917.

The authors claim that their experiments show flies to be of very great importance in the spread of amebic dysentery. They have observed the passage through the ordinary house-fly, the blue-bottle fly, and the green-bottle fly, of the living cysts of *E. coli*, *E. histolytica* and *L. intestinalis*. As E. histolytica cysts soon die if deprived of moisture it is improbable that wind plays an important part in their dissemination unless it blows about moist particles of fæces. In testing out reagents the authors found that cresol seemed the most satisfactory and was effective in a strength of 1 in 40 or 50; they therefore recommend it for the desinfection of dysenteric stools. They are of the opinion that an efficient system of fly and fæces destruction would materially reduce or eradicate amebic dysentery as well as other intestinal disorders.

